# Theoretical Framework and Guidelines for the Cyclic Voltammetry of Closed Bipolar Cells

**DOI:** 10.1021/acs.analchem.3c03480

**Published:** 2023-11-13

**Authors:** Eduardo Laborda, Javier López-Asanza, Angela Molina

**Affiliations:** Departamento de Química Física, Facultad de Química, Regional Campus of International Excellence “Campus Mare Nostrum”, Universidad de Murcia, 30100 Murcia, Spain

## Abstract

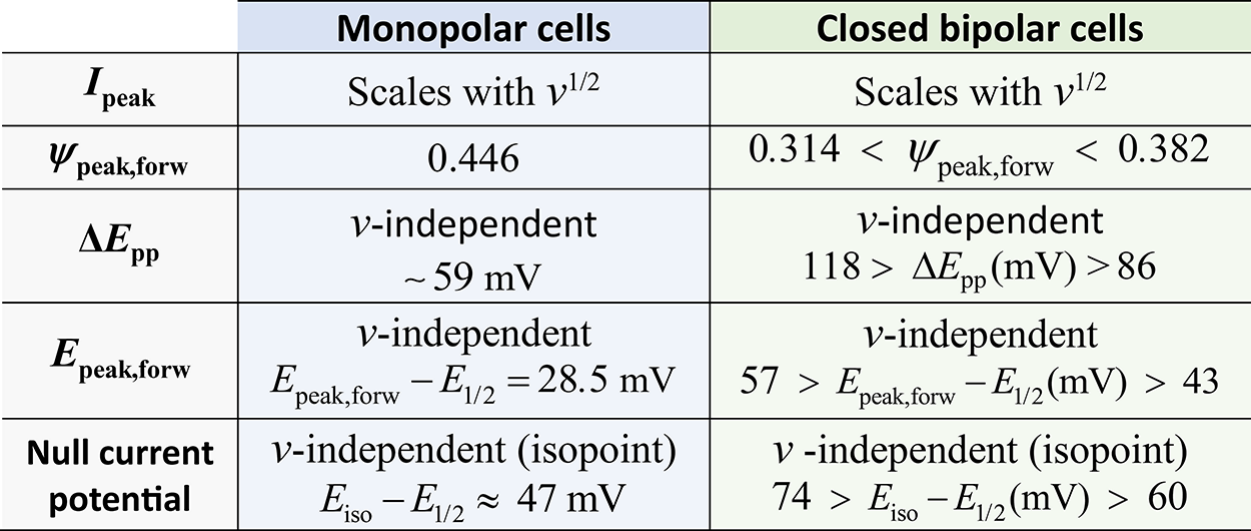

Closed bipolar cells (cBPCs) can offer valuable platforms for the development of electrochemical sensors. On the other hand, such systems are more intricate to model and interpret than conventional systems with a single polarizable interface, with the applied potential “splitting” into two polarized interfaces where two coupled charge transfers take place concomitantly. As a result, the voltammetry of cBPCs shows peculiarities that can be misleading if analyzed under the framework of classic electrochemical cells. In this work, rigorous mathematical solutions are deduced for the cyclic voltammetry (CV) of cBPCs, including the current–potential response, the interfacial potentials, and the interfacial redox concentrations. With such theoretical tools, a comprehensive view of the behavior of cBPCs can be gained, and adequate diagnosis criteria are established on the basis of the shape, magnitude, and position of the CV signal as a function of the scan rate and of the experimental conditions in the anodic and cathodic compartments.

## Introduction

Among other applications,^1,2^ closed bipolar cells (cBPCs) open advantageous approaches in electroanalysis^3−5^ by coupling the electrochemical conversion of the target species with a secondary electrochemical process that can serve as a reference or as a signal enhancer. Regarding the latter, cBPCs are employed in the efficient transduction of electrons to photons in electrogenerated chemiluniscence (ECL) systems,^6−9^ achieving very high sensitivity and spatiotemporal resolution.

**Figure 1:**
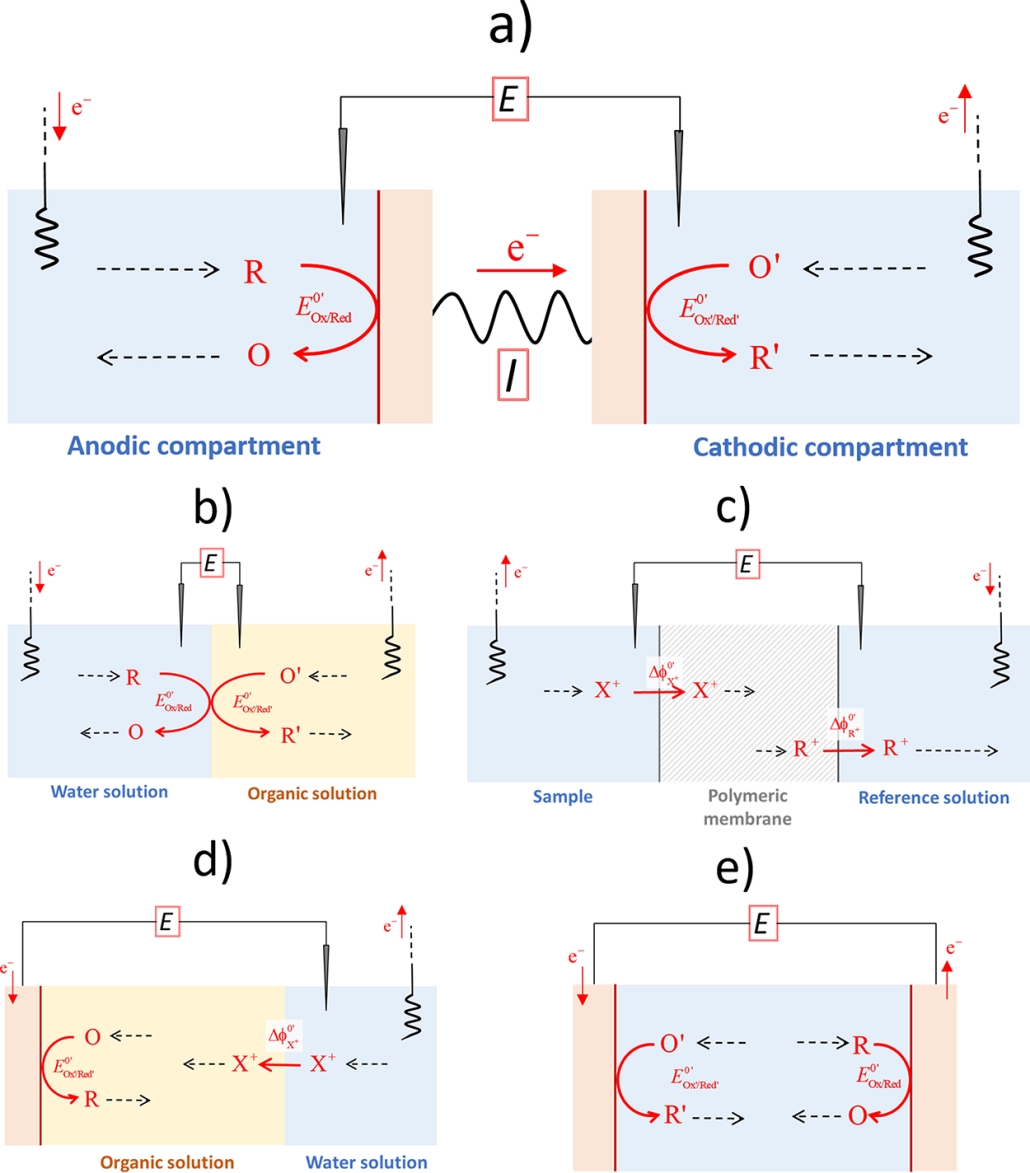
Schematics of (a) the four-electrode cBPC modeled in this work,^10,11,21,22^ together with other systems with two polarized interfaces in series and two coupled charge transfers: (b) electron transfer at a liquid–liquid interface, (c) ion transfers across a polymeric membrane, (d) coupled electron–ion transfers at film-modified electrodes, and (e) two-electrode electrolysis. In all cases, it is considered that linear diffusion is the only mass transport mechanism, and that the heterogeneous charge transfers are reversible (and monoelectronic, where appropriate).

With respect to the conventional three-electrode cell, cBPCs are more complex systems that include two polarized interfaces in series (Figure 1a), where two concomitant electron transfers take place under different interfacial potentials, which are unknown *a priori*. As a result, the voltammetry of cBPCs has been reported, mainly experimentally, to show notable differences with respect to conventional three-electrode setups. Thus, the shape and position of the current–potential signal is affected by the ratio between the bulk concentrations in the anodic and cathodic compartments,^10−12^ as well as by the relative size and geometry of the cathodic and anodic electrodes. Asymmetric conditions between the anodic and cathodic poles can also arise from different charge-transfer mechanisms and/or different mass transport conditions, for example, in the case where a catalytic mechanism operates in one of the compartments (as in ECL sensing) and/or forced convection is applied (as in alkaline water electrolysis or flow battery stacks). As a consequence of such peculiarities, the guidelines and procedures available for the qualitative and quantitative analysis of the voltammetry of three-electrode setups do not apply, and there is a need for developing an *ad hoc* theoretical framework. In this sense, the theoretical treatment of the voltammetry of cBPCs has been quite limited so far, especially in the case of multipulse techniques for which only numerical studies of the cyclic voltammetry (CV) have been reported,^11^ to the best of our knowledge.

In this work, an easily programmable, explicit analytical solution is deduced for the CV signal of two coupled reversible one-electron transfers under linear diffusion conditions in cBPCs, together with expressions for the corresponding interfacial potentials and interfacial concentrations. Altogether, they enable a deep understanding of the behavior of cBPCs and so higher capacities of prediction, optimization, and data analysis. The results here presented are also applicable to the study of coupled ion transfers at polymeric membrane sensors (Figure 1b),^13^ biphasic electron transfers at liquid–liquid interfaces (Figure 1c),^14,15^ coupled ion–electron transfers at thick film-modified electrodes (Figure 1d),^16−18^ as well as to two-electrode cyclic voltammetry^19^ and parallel paired electrolysis^20^ (Figure 1e).

On the basis of the theoretical solutions obtained, the applicability of the widely used criteria for the analysis of the CV response (that is, peak-to-peak separation, influence of scan rate on peak current, peak potential, null current potential, etc.) is revisited and conveniently adapted to the case of cBPCs, deriving procedures for the determination of concentrations and formal potentials. Similarities between the CVs of bipolar and monopolar systems are observed, both scaling with the square root of the scan rate and the signal being centered around the half-wave potential. On the other hand, significant differences are also found so that a modified Randles–Ševčík equation is necessary for the quantitative analysis of cBPCs (the peak current being always smaller), and the peak-to-peak separation is larger than in conventional cells and dependent on the experimental conditions of the two poles. Specifically, the ratio between the maximum (limiting) currents that can flow through each electrode is a chief parameter that defines the features of the CV response, guidelines being discussed in this work for the identification of the so-called “limiting” and “excess” poles.^12^

## Theory

Let us consider the four-electrode cBPC^10^ shown in Figure 1a, where the two coupled one-electron transfers are considered reversible, and the mass transport of chemical species in both compartments are due to linear diffusion. Under these conditions, the variation of the concentrations of the redox species with time (*t*) and the distance to the cathodic or anodic electrode (*x*) are given by
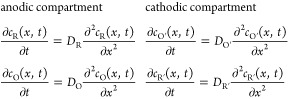
1
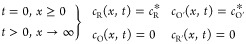
2*t* > 0, *x* = 0:
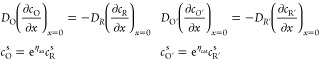
3where the dimensionless potentials η_an/cat_ are given in eq. (S4), the interfacial potentials at the two electrodes are related through the potential difference applied between the solutions in the anodic and cathodic compartments, *E*
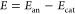
4and the current flow across the two interfaces must be the same
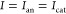
5The CV signal can be modeled considering the application of a staircase sequence of potential pulses *E*_1_, *E*_2_, ···, *E*_p_ of the same duration, τ. Considering such potential perturbation and following the procedure detailed in the Supporting Information (SI), the following expression is fully rigorous for any multipulse technique, as well as for cyclic voltammetry (CV), when the step potential is small enough (Δ*E* < 0.01 mV^23^)
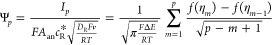
6where *v* = Δ*E*/*τ* is the scan rate (with Δ*E* being the step potential in absolute value)
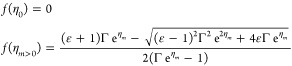
7with
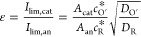
8
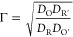
9
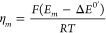
10
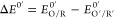
11where *I*_lim,cat_ and *I*_lim,an_ refer to the mass transport-controlled current of the cathodic and anodic electrodes, respectively
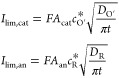
12Note that ε is a key parameter of the CV response that accounts for the relative capacity of the cathodic and anodic compartments to maintain the current flow. Unless otherwise indicated, in this work, the anodic electrode will be considered to be the limiting pole so that ε ≥ 1 according to eq 8. The opposite case, *I*_lim,cat_ < *I*_lim,an_, is evidently covered by eq 6 by just replacing ε by 1/ε, which leads to
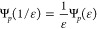
13This indicates that the voltammetric response for a given ε-value is obviously identical to that for 1/ε, once normalized with respect to the corresponding limiting pole. Note that this can be identified from the experimental values of *I*_lim,cat_ and *I*_lim,an_ recorded in a conventional three-electrode setup using the cathodic or anodic pole as the working electrode. An alternative method is discussed later.

The potential function *f*(η) can be rewritten in a more general way by referring the applied potential to the half-wave potential (see the Supporting Information)
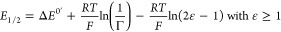
14so that
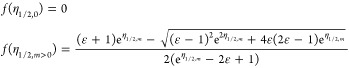
15where
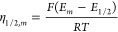
16From eq 6, it can be easily inferred that the CV curves scale with *v*^1/2^, as in the case of conventional cells, since all of the summation terms are only dependent on the applied potential and on the experimental conditions through the time-independent parameter ε.

### Particular Cases

#### Equal Conditions in the Anodic and Cathodic Compartments: ε = 1

In the simplest situation where the conditions in both compartments are the same so that ε = 1 (that is, equal electrode areas, diffusion coefficients and bulk concentrations of the redox reactants), expression (eq 7) notably simplifies to
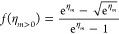
17

#### Markedly Different Conditions in the Anodic and Cathodic Compartments: ε ≫ 1

Another particular case of interest is that where the concentration of the reactant species is notably larger in one of the compartments with respect to the other: *c*_R_^*^ ≫ *c*_O′_^*^. This limit situation coincides with that of two coupled ion transfers at polymeric membranes sensors, where the nontarget ion is frequently present in a large excess.^24^ Under these conditions, a simplified mathematical solution is deduced by making ε ≫ 1 so that eq 7 becomes
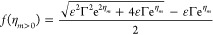
18The values obtained from eq 18 differ less than 1% from the general expression (eq 7) when ε > 20.

#### Electron Transfers at Liquid–Liquid Interfaces

It is worth noting that the analytical solution (eq 6) also applies to the case of electron transfers at liquid–liquid interfaces (Figure 1c),^14,15^ just by taking into account that the hypothetical anodic and cathodic electrodes have the same area: *A*_cat_ = *A*_an_ so that .

## Results and Discussion

**Figure 2:**
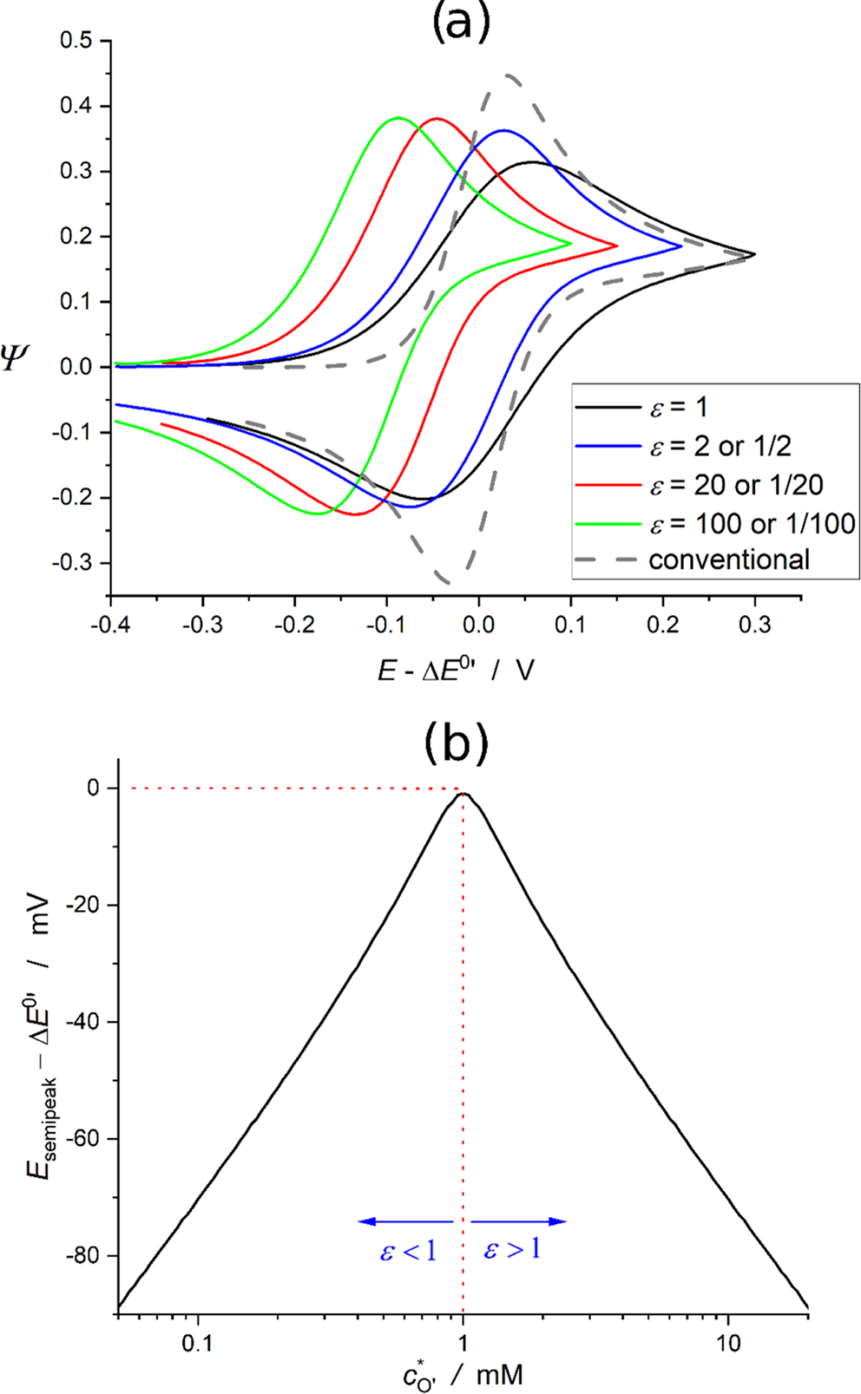
Cyclic voltammetry (eq 6) of the cBPC considered in Figure 1a for different values of the parameter ε (eq 8), together with the CV response in conventional monopolar cells (gray dashed line). *D*_O_=*D*_R_, *D*_O'_=*D*_R'_, Δ*E* = 0.01 mV, *T* = 298 K. In Figure 2a,  for ε ≥ 1 and  for ε ≤ 1.

Figure 2a shows the semidimensionless CV response at cBPCs as a function of the value of ε. For the sake of comparison, the response at a conventional three-electrode setup is also included (dashed gray line).

In the case where both compartments have the same conditions for the flow of current, ε = 1 (black solid line), the CV signal is centered around the value Δ*E*^0′^, and it shows a peak-to-peak separation (Δ*E*_pp_) of Δ*E*_pp_(ε = 1) ≈ 118 mV, which doubles the well-known value of reversible one-electron transfers in monopolar cells.^23,25−27^ The dimensionless peak current of the forward scan (Ψ_peak,forw_) also differs significantly from the value predicted by the Randles–Ševčík equation (i.e., Ψ_peak,forw_ = 0.446), being smaller by a factor of :^27^ Ψ_peak,forw_(ε = 1) = 0.314.

When the conditions of the two compartments are different, the position, height, and peak-to-peak separation of the CV response change. As ε differs more from unity, there is a continuous shift of the signal toward more negative values, as well as an increase of the peak current and a decrease of Δ*E*_pp_ up to reaching the limit values of Ψ_peak,forw_(ε ≫ 1) = 0.382 and Δ*E*_pp_(ε ≫ 1) = 86 mV for very large (or very small) ε-values.

For intermediate situations, the following relationships are found to provide an accurate description of the variation of Ψ_peak,forw_ and Δ*E*_pp_ with ε (accuracy better than 1% and 1 mV, respectively)
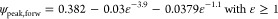
19
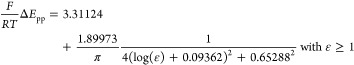
20Note that, as anticipated from eq 13, upon appropriate normalization (see caption of Figure 2), the CV signal of any given ε-value is identical to that of 1/ε (see Figure 2a). Hence, the peak-to-peak separation and the position of the voltammogram are the same for ε and 1/ε, that is, they are dependent on the magnitude of the asymmetry between the maximum current flows of the anodic and the cathodic compartments, regardless of which one of them acts as the limiting pole.

In practice, the value of ε varies with the area of the electrodes and with the diffusion coefficient and bulk concentrations of the redox species (see eq 8). For example, let us consider the influence of the concentration of species O′ in the cathodic compartment on the CV response of a cBPC, where the concentration of R in the anodic solution is maintained unaltered. As shown in Figure 2b for the plausible conditions , the position of the CV curve is always sensitive to the value of *c*_O′_^*^ in such a way that *E*_semipeak_ = (*E*_peak,forw_ + *E*_peak,back_)/2 will shift toward less negative potentials with the increase of *c*_O′_^*^ when the cathodic compartment is the limiting pole (ε < 1, *c*_O′_^*^ < 1 mM in Figure 2b), while it will shift toward more negative potentials when the cathodic compartment is the excess pole (ε > 1, *c*_O′_^*^ > 1 mM). The maximum *E*_semipeak_-value is ca. Δ*E*^0′^, which will be found when ε = 1 (*c*_O′_^*^ = *c*_R_^*^ in Figure 2b).

The above behaviors of the CV signal can be useful for the characterization of the cBPC or of an unknown solution. Regarding the former, by intentionally varying the reactant concentration in one of the compartments, the resulting shift of the signal will reveal whether such a compartment corresponds to the limiting or the excess pole. Alternatively, by using a well-characterized cBPC (i.e., known *A*_an_ and *A*_cat_) with a reference solution in one of the compartments (for example, the anodic compartment so that *c*_R_^*^, *D*_R_, and *E*_O/R_^0′^ are known), ψ_peak,forw_ can be calculated from the experimental peak current, and then the ε-value can be solved from eq 19.^a^ Once ε is known, the target concentration can be immediately calculated as *c*_O′_^*^ = ε*c*_R_^*^A_an_/*A*_cat_ (assuming similar diffusivity for O′ and R). Finally, the formal potential of the redox couple of O′/R′ can be extracted from *E*_semipeak_, taking into account that its value is close to the half-wave potential (eq 14), as in conventional cells.

**Figure 3:**
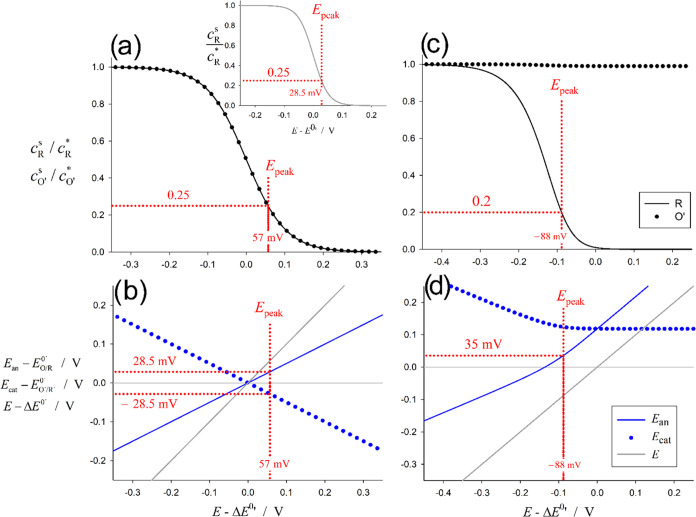
Variation of the interfacial concentrations of the redox reactants and the interfacial potentials (eqs (S58)) along the forward CV scan for (a, c) ε = 1 and (b, d) ε = 100. (Inset) Variation of the reactant’s surface concentration in a conventional monopolar cell. Other conditions are as in Figure 2.

The underlying origin of the differences between the CV in monopolar and bipolar cells is investigated in Figure 3 through the study of the variation of the interfacial concentrations and the interfacial potentials along the forward scan. As shown in Figure 3b, when ε = 1 the applied potential “splits” equally into the anodic and cathodic interfaces in such a way that the depletion of the reactants R and O′ takes place at more positive *E*-values (*E*_peak_ – Δ*E*^0′^ = 57 mV) than in conventional cells (*E*_peak_ – *E*^0′^ = 28.5 mV^25^). This means that the peak in cBPCs is observed at a longer perturbation time, when the diffusion layer is larger, and so the surface concentration gradient and the peak current are smaller. Looking in more detail, it is found that the forward peak in cBPCs with ε = 1 corresponds to the *E*-value where *E*_an_ – *E*_O/R_^0′^ = 28.5 mV, *E*_cat_ – *E*_O′R′_^0′^ = −28.5 mV, and the interfacial concentrations of R and O′ has dropped to 25% their initial values, which is totally equivalent to what happens in systems with a single polarizable interface (see the inset in Figure 3a).

The case ε ≫ 1 is considered in Figure 3c,3d where ε = 100. Under such conditions, the applied driving force is mainly “consumed” by the anodic limiting pole, with the potential difference at the cathodic excess pole remaining at positive values and almost constant. Thus, *E*_an_ – *E*_O/R_^0′^ soon reaches positive values (namely, at *E* – Δ*E*^0′^ ≃ -135 mV vs *E* – Δ*E*^0′^ = 0 mV for ε = 1, compare Figure 3b,3d) and the conditions for the appearance of the voltammetric peak, which are close (though not identical) to those for ε = 1, specifically: *E*_an_ – *E*_O/R_^0′^ = 35 mV and *c*_R_^s^ = 0.2*c*_R_^*^.

**Figure 4:**
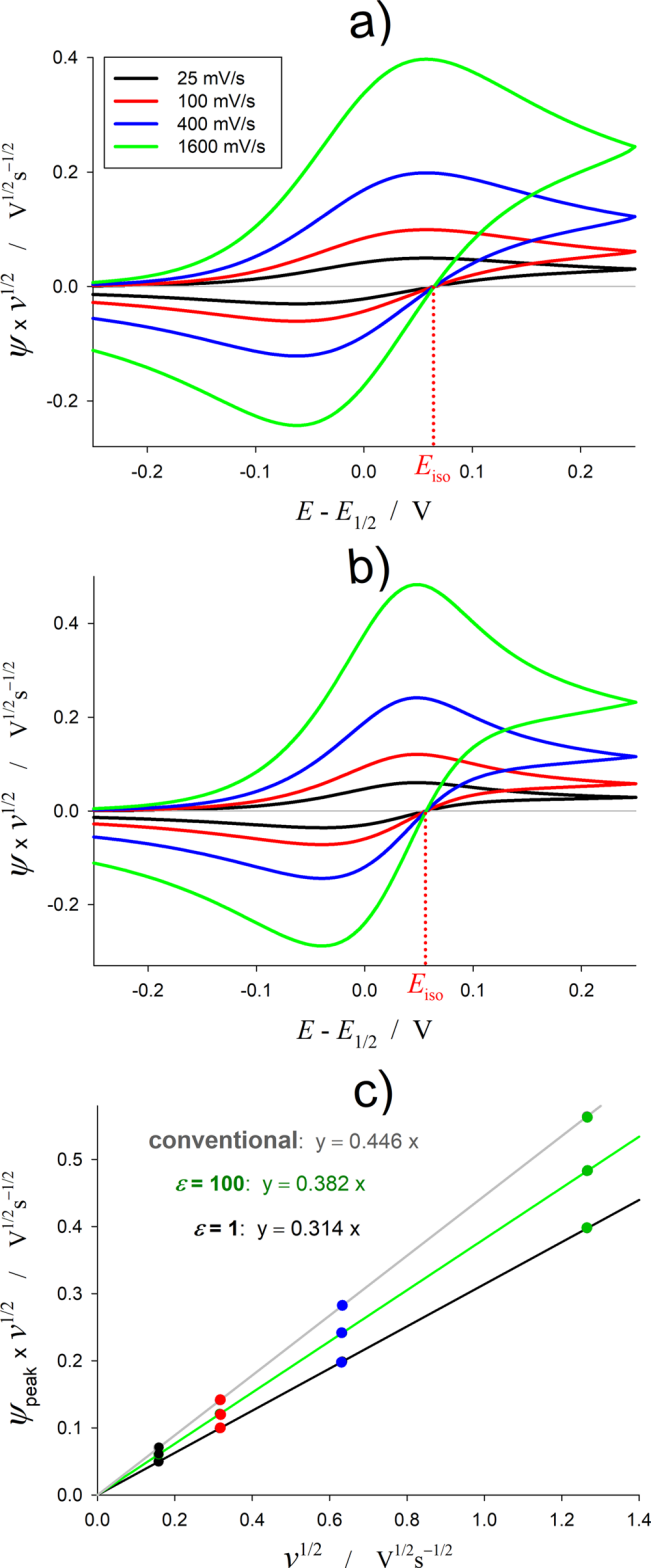
Influence of the scan rate, *v*, on the CV response of cBPCs for (a) ε = 1, (b) ε = 100, and (c) on the forward peak current. Other conditions are as in Figure 2.

The cyclic voltammetry of cBPCs also has similarities with the response of conventional cells. With respect to the influence of the scan rate, as shown in Figure 4 and as can be directly deduced from eq 6, the forward peak current (*I*_peak,forw_) scales with the square root of the scan rate, regardless of the ε-value. Nevertheless, the slope of the plot *I*_peak,forw_ vs *v*^1/2^ does depend on ε: the more different from unity the ε-value, the larger the slope, being always smaller (between ca. 15 and 30%) than the slope of monopolar systems. Hence, the use of the Randles–Ševčík equation for cBPCs would yield significant underestimations of the electrode area, the diffusion coefficient, or the bulk concentration of redox species. With regard to the peak potential and the peak-to-peak separation, as in the case of conventional three-electrode systems, they do not change with the scan rate^11^ so that the shape and position of the CV signal is independent of *v*. It is also worth noting the existence of a null current point (the so-called isopoint^23,28^), independent of the scan rate for any ε-value. This is characteristic of semi-infinite linear diffusion conditions and enables the determination of ε or the half-wave potential
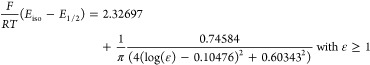
21

## Conclusions

Rigorous and manageable mathematical expressions have been deduced for the current–potential response, interfacial concentrations, and interfacial potentials of the cyclic voltammetry (CV) of closed bipolar cells (cBPCs), enabling the comprehensive analysis of such systems as well as others with two coupled heterogeneous charge transfers.

The theory points out that the chief parameter of the CV response is the ratio between the limiting currents of the cathodic and anodic poles: . Thus, different values of ε mean different distributions of the applied potential between the anodic and cathodic interfaces, which affects the interfacial concentrations of the redox species and the features of the cyclic voltammograms. These show some remarkable differences with respect to the CV of conventional cells with a single polarizable interface: the peak current (*I*_peak_) is 15–30% smaller in cBPCs, the peak-to-peak separation (Δ*E*_pp_) is 1.5–2 times larger, and the signal position (*E*_semipeak_ ≃ *E*_1/2_) changes with the concentration of reactants. Rigorous or very accurate mathematical relationships for *I*_peak_, Δ*E*_pp_, and *E*_1/2_ as a function of ε have been presented, which allow for the identification and characterization of the limiting and excess poles or for the investigation of a target redox species using one of the compartments as an internal reference.

## Supporting Information Available

Mathematical derivation of the analytical theory for voltammetric response of closed bipolar cells (PDF)

## Footnotes

a Note that if the calculated ψ_peak,forw_-value is smaller than 0.314, this will mean that the experimental situation corresponds to ε < 1, for which, according to eqs 13, 14, and 19, it is fulfilled that ψ_peak,forw_ = 0.382ε – 0.03ε^4.9^ – 0.0379ε^2.1^ and .
