# Supplementary material for: Theoretical Framework and Guidelines for the Cyclic Voltammetry of Closed Bipolar Cells
Source: Anal Chem. 2023 Nov 13;95(47):17311–7. doi: 10.1021/acs.analchem.3c03480 (PMC10688229; doi:10.1021/acs.analchem.3c03480)
Supplement: Supplementary file 1 — ac3c03480_si_001.pdf [file ac3c03480_si_001.pdf]

SUPPORTING INFORMATION

Theoretical Framework and Guidelines for the Cyclic Voltammetry of Closed Bipolar Cells

Eduardo Laborda*, Javier López-Asanza, Angela Molina*

Departamento de Química Física, Facultad de Química, Regional Campus of International Excellence "Campus Mare Nostrum", Universidad de Murcia, 30100 Murcia, Spain

* Corresponding authors:

Tel: +34 868 88 7524; 7433

Fax: +34 868 88 4148

Email: amolina@um.es; elaborda@um.es

TABLE OF CONTENTS

SI. Analytical theory for voltammetric response of closed bipolar cells (cBPC's)

SI1. Constant potential pulse

SI2. Second potential pulse

SI3. Any p-th potential pulse

SI. Analytical theory for voltammetric response of closed bipolar cells (cBPC's)

SI1. Constant potential pulse

The boundary value problem (bvp) corresponding to the cBPC shown Figure 1a of the main text when a constant potential pulse, *E*, is applied is given by:

$$
\begin{array}{ll}
\text{Anodic compartment} & \text{Cathodic compartment} \\
\dfrac{\partial c_{\mathrm{R}}(x,t)}{\partial t} = D_{\mathrm{R}} \dfrac{\partial^2 c_{\mathrm{R}}(x,t)}{\partial x^2} & \dfrac{\partial c_{\mathrm{O'}}(x,t)}{\partial t} = D_{\mathrm{O'}} \dfrac{\partial^2 c_{\mathrm{O'}}(x,t)}{\partial x^2} \\
\dfrac{\partial c_{\mathrm{O}}(x,t)}{\partial t} = D_{\mathrm{O}} \dfrac{\partial^2 c_{\mathrm{O}}(x,t)}{\partial x^2} & \dfrac{\partial c_{\mathrm{R'}}(x,t)}{\partial t} = D_{\mathrm{R'}} \dfrac{\partial^2 c_{\mathrm{R'}}(x,t)}{\partial x^2}
\end{array} \tag{S1}
$$

$$
\left.\begin{array}{l} t=0,\ x\geq 0 \\ t>0,\ x\to\infty \end{array}\right\} \quad
\begin{array}{ll}
c_{\mathrm{R}}(x,t) = c_{\mathrm{R}}^{*} & c_{\mathrm{O'}}(x,t) = c_{\mathrm{O'}}^{*} \\
c_{\mathrm{O}}(x,t) = 0 & c_{\mathrm{R'}}(x,t) = 0
\end{array} \tag{S2}
$$

$t>0, x=0$:

$$
\begin{array}{ll}
D_{\mathrm{O}}\left(\dfrac{\partial c_{\mathrm{O}}}{\partial x}\right)_{x=0} = -D_{\mathrm{R}}\left(\dfrac{\partial c_{\mathrm{R}}}{\partial x}\right)_{x=0} & D_{\mathrm{O'}}\left(\dfrac{\partial c_{\mathrm{O'}}}{\partial x}\right)_{x=0} = -D_{\mathrm{R'}}\left(\dfrac{\partial c_{\mathrm{R'}}}{\partial x}\right)_{x=0} \\
c_{\mathrm{O}}^{\mathrm{s}} = \mathrm{e}^{\eta_{\mathrm{an}}} c_{\mathrm{R}}^{\mathrm{s}} & c_{\mathrm{O'}}^{\mathrm{s}} = \mathrm{e}^{\eta_{\mathrm{cat}}} c_{\mathrm{R'}}^{\mathrm{s}}
\end{array} \tag{S3}
$$

with $c_{\mathrm{i}}^{\mathrm{s}}$ being the interfacial concentration of species i $(\equiv \mathrm{O, R, O', R'})$ and:

$$
\begin{array}{l}
\eta_{\mathrm{an}} = \dfrac{F}{RT}\left(E_{\mathrm{an}} - E_{\mathrm{O/R}}^{0'}\right) \\
\eta_{\mathrm{cat}} = \dfrac{F}{RT}\left(E_{\mathrm{cat}} - E_{\mathrm{O'/R'}}^{0'}\right)
\end{array} \tag{S4}
$$

where the unknown potential differences at the anodic and cathodic electrode-solution interfaces are related by:

$$E = E_{\mathrm{an}} - E_{\mathrm{cat}} \tag{S5}$$

and the current across the two interfaces must be equal:

$$I = I_{\mathrm{an}} = I_{\mathrm{cat}} \tag{S6}$$

For the above problem, it is convenient to introduce the following variable:

$$s_{\mathrm{i}} = \frac{x}{2\sqrt{D_{\mathrm{i}}\,t}} \quad (\mathrm{i} \equiv \mathrm{O, R, O', R'}) \tag{S7}$$

so that the bvp becomes into:

$$
\begin{array}{ll}
\text{Anodic compartment} & \text{Cathodic compartment} \\
\dfrac{\mathrm{d}^2 c_{\mathrm{R}}(s_{\mathrm{R}})}{\mathrm{d}s_{\mathrm{R}}^{\ 2}} + 2\mathrm{s}_{\mathrm{R}} \dfrac{\mathrm{d}c_{\mathrm{R}}(s_{\mathrm{R}})}{\mathrm{d}s_{\mathrm{R}}} = 0 & \dfrac{\mathrm{d}^2 c_{\mathrm{R'}}(s_{\mathrm{R'}})}{\mathrm{d}s_{\mathrm{R'}}^{\ 2}} + 2\mathrm{s}_{\mathrm{R'}} \dfrac{\mathrm{d}c_{\mathrm{R'}}(s_{\mathrm{R'}})}{\mathrm{d}s_{\mathrm{R'}}} = 0 \\
\dfrac{\mathrm{d}^2 c_{\mathrm{O}}(s_{\mathrm{O}})}{\mathrm{d}s_{\mathrm{O}}^{\ 2}} + 2\mathrm{s}_{\mathrm{O}} \dfrac{\mathrm{d}c_{O}(s_{\mathrm{O}})}{\mathrm{d}s_{\mathrm{O}}} = 0 & \dfrac{\mathrm{d}^2 c_{\mathrm{O'}}(s_{\mathrm{O'}})}{\mathrm{d}s_{\mathrm{O'}}^{\ 2}} + 2\mathrm{s}_{\mathrm{O'}} \dfrac{\mathrm{d}c_{O'}(s_{\mathrm{O'}})}{\mathrm{d}s_{\mathrm{O'}}} = 0
\end{array} \tag{S8}
$$

$$s_i \to \infty\} \qquad c_{\rm R}(s_{\rm R}) = c_{\rm R}^* \qquad c_{\rm O'}(s_{\rm O'}) = c_{\rm O'}^*$$
$$c_{\rm O}(s_{\rm O}) = 0 \qquad c_{\rm R'}(s_{\rm R'}) = 0 \tag{S9}$$

$s_i = 0$:

$$\sqrt{D_{\rm O}}\left(\frac{\partial c_{\rm O}}{\partial s_{\rm O}}\right)_{s_{\rm O}=0} = -\sqrt{D_{\rm R}}\left(\frac{\partial c_{\rm R}}{\partial s_{\rm R}}\right)_{s_{\rm R}=0} \qquad \sqrt{D_{\rm O'}}\left(\frac{\partial c_{\rm O'}}{\partial s_{\rm O'}}\right)_{s_{\rm O'}=0} = -\sqrt{D_{\rm R'}}\left(\frac{\partial c_{\rm R'}}{\partial s_{\rm R'}}\right)_{s_{\rm R'}=0}$$
$$c_{\rm O}^{\rm s} = {\rm e}^{\eta_{\rm an}} c_{\rm R}^{\rm s} \qquad c_{\rm O'}^{\rm s} = {\rm e}^{\eta_{\rm cat}} c_{\rm R'}^{\rm s} \tag{S10}$$

This problem can be easily solved by defining $y_{\rm i} = {\rm d}c_{\rm i}/{\rm d}s_{\rm i}$ $({\rm i} \equiv {\rm O, R, O', R'})$, such that equations (S8) become:

$$\frac{{\rm d}y_{\rm i}}{{\rm d}s_{\rm i}} + 2{\rm s_i}y_{\rm i} = 0 \tag{S11}$$

the double integration of which, with the application of the initial and bulk conditions (S9) [1], leads to:

Anodic compartment / Cathodic compartment

$$c_{\rm R} = c_{\rm R}^* + (c_{\rm R}^{\rm s} - c_{\rm R}^*)\,{\rm erfc}\left(\frac{x}{2\sqrt{D_{\rm R}t}}\right) \qquad c_{\rm R'} = c_{\rm R'}^{\rm s}\,{\rm erfc}\left(\frac{x}{2\sqrt{D_{\rm R'}t}}\right) \tag{S12}$$
$$c_{\rm O} = c_{\rm O}^{\rm s}\,{\rm erfc}\left(\frac{x}{2\sqrt{D_{\rm O}t}}\right) \qquad c_{\rm O'} = c_{\rm O'}^* + (c_{\rm O'}^{\rm s} - c_{\rm O'}^*)\,{\rm erfc}\left(\frac{x}{2\sqrt{D_{\rm O'}t}}\right)$$

Taking into account eqns. (S12) and the relationships between the current at the anodic and cathodic poles and the surface gradients of the redox species:

$$I_{\rm an}\left(=I\right) = FA_{\rm an}D_{\rm R}\left(\frac{\partial c_{\rm R}}{\partial x}\right)_{x=0} = -FA_{\rm an}D_{\rm O}\left(\frac{\partial c_{\rm O}}{\partial x}\right)_{x=0} \tag{S13}$$

$$I_{\rm cat}\left(=I\right) = FA_{\rm cat}D_{\rm O'}\left(\frac{\partial c_{\rm O'}}{\partial x}\right)_{x=0} = -FA_{\rm cat}D_{\rm R'}\left(\frac{\partial c_{\rm R'}}{\partial x}\right)_{x=0} \tag{S14}$$

the following relationships can be immediately deduced between the interfacial concentrations and the current:

Anodic compartment / Cathodic compartment

$$c_{\rm R}^{\rm s} = c_{\rm R}^*(1 - I_{\rm N}) \qquad c_{\rm R'}^{\rm s} = c_{\rm O'}^*\frac{I_{\rm N}\gamma'}{\varepsilon} \tag{S15}$$
$$c_{\rm O}^{\rm s} = c_{\rm R}^*\frac{I_{\rm N}}{\gamma} \qquad c_{\rm O'}^{\rm s} = c_{\rm O'}^*\left(1 - \frac{I_{\rm N}}{\varepsilon}\right)$$

where:

$$I_{\mathrm{N}} = \frac{I}{I_{\mathrm{lim,an}}} \tag{S16}$$

$$\gamma = \sqrt{\frac{D_{\mathrm{O}}}{D_{\mathrm{R}}}} \tag{S17}$$

$$\gamma' = \sqrt{\frac{D_{\mathrm{O'}}}{D_{\mathrm{R'}}}} \tag{S18}$$

$$\varepsilon = \frac{I_{\mathrm{lim},cat}}{I_{\mathrm{lim},an}} = \frac{A_{\mathrm{cat}} c_{\mathrm{O'}}^*}{A_{\mathrm{an}} c_{\mathrm{R}}^*} \sqrt{\frac{D_{\mathrm{O'}}}{D_{\mathrm{R}}}} \tag{S19}$$

with $I_{\mathrm{lim,an}}$ and $I_{\mathrm{lim,cat}}$ being the limiting current of the cathodic and anodic electrodes, respectively [1]:

$$\begin{aligned} I_{\mathrm{lim,cat}} &= F A_{\mathrm{cat}} c_{\mathrm{O'}}^* \sqrt{\frac{D_{\mathrm{O'}}}{\pi t}} \\ I_{\mathrm{lim,an}} &= F A_{\mathrm{an}} c_{\mathrm{R}}^* \sqrt{\frac{D_{\mathrm{R}}}{\pi t}} \end{aligned} \tag{S20}$$

Combining the Nernstian conditions in (S10) with equations (S15), the following expressions are obtained for the interfacial potentials:

$$E_{\mathrm{an}} = E_{\mathrm{O/R}}^{0'} + \frac{RT}{F} \ln\left(\frac{I_{\mathrm{N}}}{(1 - I_{\mathrm{N}})\gamma} \right) \tag{S21}$$

$$E_{\mathrm{cat}} = E_{\mathrm{O'/R'}}^{0'} + \frac{RT}{F} \ln\left(\frac{\varepsilon - I_{\mathrm{N}}}{\gamma' I_{\mathrm{N}}} \right) \tag{S22}$$

Taking into account eqn. (S5), the following relationship between the applied potential and the current is deduced:

$$E = E_{\mathrm{an}} - E_{\mathrm{cat}} = \Delta E^{0'} + \frac{RT}{F} \ln\left(\frac{I_{\mathrm{N}}^2}{\left(\varepsilon - I_{\mathrm{N}}\right)\left(1 - I_{\mathrm{N}}\right)\Gamma} \right) \tag{S23}$$

with

$$\Delta E^{0'} = E_{\mathrm{O/R}}^{0'} - E_{\mathrm{O'/R'}}^{0'} \tag{S24}$$

$$\Gamma = \frac{\gamma}{\gamma'} \tag{S25}$$

From expression (S23), the value of the half-wave potential, $E_{1/2}$, is immediately extracted by imposing the condition $I_{\mathrm{N}} = 1/2$:

$$E_{1/2} = \Delta E^{0'} + \frac{RT}{F} \ln\left(\frac{1}{\Gamma} \right) - \frac{RT}{F} \ln\left(2\varepsilon - 1\right) \quad \text{with} \quad \varepsilon \geq 1 \tag{S26}$$

Finally, solving the current in eqn. (S23), the following expression for the *I-E* curve is obtained:

$$I = FA_{\text{an}} c_{\text{R}}^{*} \sqrt{\frac{D_{\text{R}}}{\pi t}}\, f(\eta) \tag{S27}$$

where:

$$f(\eta) = \frac{\left(\varepsilon+1\right)\Gamma \mathrm{e}^{\eta} - \sqrt{\left(\varepsilon-1\right)^{2} \Gamma^{2} \mathrm{e}^{2\eta} + 4\,\varepsilon\,\Gamma e^{\eta}}}{2\left(\Gamma e^{\eta}-1\right)} \tag{S28}$$

with

$$\eta = \frac{F(E-\Delta E^{0'})}{RT} \tag{S29}$$

Note that, taking into account solution (S27), the interfacial potentials and concentrations can be re-written in terms of the applied potential as follows:

$$\begin{array}{ll} \text{Anodic compartment} & \text{Cathodic compartment} \\ c_{\text{R}}^{\text{s}} = c_{\text{R}}^{*}\left(1-f(\eta)\right) & c_{\text{R'}}^{\text{s}} = c_{\text{O'}}^{*}\dfrac{f(\eta)\gamma'}{\varepsilon} \\ c_{\text{O}}^{\text{s}} = c_{\text{R}}^{*}\dfrac{f(\eta)}{\gamma} & c_{\text{O'}}^{\text{s}} = c_{\text{O'}}^{*}\left(1-\dfrac{f(\eta)}{\varepsilon}\right) \\ E_{\text{an}} = E_{\text{O/R}}^{0'} + \dfrac{RT}{F}\ln\left(\dfrac{f(\eta)}{\left(1-f(\eta)\right)\gamma}\right) & E_{\text{cat}} = E_{\text{O'/R'}}^{0'} + \dfrac{RT}{F}\ln\left(\dfrac{\varepsilon-f(\eta)}{\gamma' f(\eta)}\right) \end{array} \tag{S30}$$

SI2. Second potential pulse

Let us consider the application of a second potential pulse, E_2, at $t=\tau_1$ for the period of time $\tau_1 \le t \le \tau_1+\tau_2$ so that τ_1 and τ_2 are the duration of the first and second pulse, respectively. The bvp of the second pulse is given by:

$$\begin{array}{ll} \text{Anodic compartment} & \text{Cathodic compartment} \\ \dfrac{\partial c_{\text{R}}^{(2)}(x,t)}{\partial t} = D_{\text{R}}\dfrac{\partial^2 c_{\text{R}}^{(2)}(x,t)}{\partial x^2} & \dfrac{\partial c_{\text{O'}}^{(2)}(x,t)}{\partial t} = D_{\text{O'}}\dfrac{\partial^2 c_{\text{O'}}^{(2)}(x,t)}{\partial x^2} \\ \dfrac{\partial c_{\text{O}}^{(2)}(x,t)}{\partial t} = D_{\text{O}}\dfrac{\partial^2 c_{\text{O}}^{(2)}(x,t)}{\partial x^2} & \dfrac{\partial c_{\text{R'}}^{(2)}(x,t)}{\partial t} = D_{\text{R'}}\dfrac{\partial^2 c_{\text{R'}}^{(2)}(x,t)}{\partial x^2} \end{array} \tag{S31}$$

$$\left.\begin{array}{l} t=\tau_1,\ x\ge 0 \\ t>\tau_1,\ x\to\infty \end{array}\right\} \quad \begin{array}{ll} c_{\text{R}}^{(2)} = c_{\text{R}}^{(1)} & c_{\text{O'}}^{(2)} = c_{\text{O'}}^{(1)} \\ c_{\text{O}}^{(2)} = c_{\text{O}}^{(1)} & c_{\text{R'}}^{(2)} = c_{\text{R'}}^{(1)} \end{array} \tag{S32}$$

$t > \tau_1$, $x = 0$:

$$D_{\text{O}}\left(\frac{\partial c_{\text{O}}^{(2)}}{\partial x}\right)_{x=0} = -D_{\text{R}}\left(\frac{\partial c_{\text{R}}^{(2)}}{\partial x}\right)_{x=0} \qquad D_{\text{O'}}\left(\frac{\partial c_{\text{O'}}^{(2)}}{\partial x}\right)_{x=0} = -D_{\text{R'}}\left(\frac{\partial c_{\text{R'}}^{(2)}}{\partial x}\right)_{x=0} \tag{S33}$$
$$c_{\text{O}}^{\text{s}(2)} = \text{e}^{\eta_{\text{an}}} c_{\text{R}}^{\text{s}(2)} \qquad c_{\text{O'}}^{\text{s}(2)} = \text{e}^{\eta_{\text{cat}}} c_{\text{R'}}^{\text{s}(2)}$$

with

$$E_2 = E_{2,\text{an}} - E_{2,\text{cat}} \tag{S34}$$

$$I_2 = I_{2,\text{cat}} = I_{2,\text{an}} \tag{S35}$$

$c_{\text{i}}^{(1)}(x,t)$ $(\text{i} \equiv \text{O, R, O', R'})$ being the solutions of the first potential pulse (eqns. (S12)) and:

$$\eta_{2,\text{an}} = \frac{F}{RT}(E_{2,\text{an}} - E_{\text{O/R}}^{0'}) \qquad \eta_{2,\text{cat}} = \frac{F}{RT}(E_{2,\text{cat}} - E_{\text{O'/R'}}^{0'}) \tag{S36}$$

Taking into account that the diffusion operator in (S1) is linear, the solutions corresponding to the second potential pulse can be written as linear combinations of solutions:

Anodic compartment / Cathodic compartment

$$c_{\text{R}}^{(2)}(x,t) = c_{\text{R}}^{(1)}(x,t) + \tilde{c}_{\text{R}}^{(2)}(x,t_2) \qquad c_{\text{R'}}^{(2)}(x,t) = c_{\text{R'}}^{(1)}(x,t) + \tilde{c}_{\text{R'}}^{(2)}(x,t_2) \tag{S37}$$
$$c_{\text{O}}^{(2)}(x,t) = c_{\text{O}}^{(1)}(x,t) + \tilde{c}_{\text{O}}^{(2)}(x,t_2) \qquad c_{\text{O'}}^{(2)}(x,t) = c_{\text{O'}}^{(1)}(x,t) + \tilde{c}_{\text{O'}}^{(2)}(x,t_2)$$

where $t_2 = t - \tau_1$ (so that $0 \le t_2 \le \tau_2$) and $\tilde{c}_{\text{i}}^{(2)}(x,t_2)$ are the unknown partial solutions of the bvp resulting from introducing eqns. (S37) into (S31)-(S33):

Anodic compartment / Cathodic compartment

$$\frac{\partial \tilde{c}_{\text{R}}^{(2)}(x,t_2)}{\partial t_2} = D_{\text{R}} \frac{\partial^2 \tilde{c}_{\text{R}}^{(2)}(x,t_2)}{\partial x^2} \qquad \frac{\partial \tilde{c}_{\text{O'}}^{(2)}(x,t_2)}{\partial t_2} = D_{\text{O'}} \frac{\partial^2 \tilde{c}_{\text{O'}}^{(2)}(x,t_2)}{\partial x^2} \tag{S38}$$
$$\frac{\partial \tilde{c}_{\text{O}}^{(2)}(x,t_2)}{\partial t_2} = D_{\text{O}} \frac{\partial^2 \tilde{c}_{\text{O}}^{(2)}(x,t_2)}{\partial x^2} \qquad \frac{\partial \tilde{c}_{\text{R'}}^{(2)}(x,t_2)}{\partial t} = D_{\text{R'}} \frac{\partial^2 \tilde{c}_{\text{R'}}^{(2)}(x,t_2)}{\partial x^2}$$

$$\left.\begin{matrix} t_2 = 0, \ x \ge 0 \\ t_2 > 0, \ x \to \infty \end{matrix}\right\} \qquad \begin{matrix} \tilde{c}_{\text{R}}^{(2)} = 0 \\ \tilde{c}_{\text{O}}^{(2)} = 0 \end{matrix} \qquad \begin{matrix} \tilde{c}_{\text{O'}}^{(2)} = 0 \\ \tilde{c}_{\text{R'}}^{(2)} = 0 \end{matrix} \tag{S39}$$

$t_2 > 0, x = 0$:

$$D_{\text{O}}\left(\frac{\partial \tilde{c}_{\text{O}}^{(2)}}{\partial x}\right)_{x=0} = -D_{\text{R}}\left(\frac{\partial \tilde{c}_{\text{R}}^{(2)}}{\partial x}\right)_{x=0} \qquad D_{\text{O'}}\left(\frac{\partial \tilde{c}_{\text{O'}}^{(2)}}{\partial x}\right)_{x=0} = -D_{\text{R'}}\left(\frac{\partial \tilde{c}_{\text{R'}}^{(2)}}{\partial x}\right)_{x=0} \tag{S40}$$
$$\tilde{c}_{\text{O}}^{\text{s}(2)} = \text{e}^{\eta_{2,\text{an}}} \tilde{c}_{\text{R}}^{\text{s}(2)} + \left(\text{e}^{\eta_{2,\text{an}}} c_{\text{R}}^{\text{s}(1)} - c_{\text{O}}^{\text{s}(1)}\right) \qquad \tilde{c}_{\text{O'}}^{\text{s}(2)} = \text{e}^{\eta_{2,\text{cat}}} \tilde{c}_{\text{R'}}^{\text{s}(2)} + \left(\text{e}^{\eta_{2,\text{cat}}} c_{\text{R'}}^{\text{s}(1)} - c_{\text{O'}}^{\text{s}(1)}\right)$$

As the values of $c_{\rm i}^{\rm s(1)}$ $({\rm i} \equiv {\rm O, R, O', R'})$ are independent of time (eqns. (S30)), the mathematical problem of $\tilde{c}_{\rm i}^{(2)}(x,t_2)$ is formally identical to that of the first pulse with null initial conditions. Hence, the solutions of $\tilde{c}_{\rm i}^{(2)}(x,t_2)$ have the following form:

Anodic compartment — Cathodic compartment

$$\tilde{c}_{\rm R}^{(2)}(x,t_2) = \tilde{c}_{\rm R}^{\rm s(2)}\,{\rm erfc}\left(\frac{x}{2\sqrt{D_{\rm R}t_2}}\right) \qquad \tilde{c}_{\rm R'}^{(2)}(x,t_2) = \tilde{c}_{\rm R'}^{\rm s(2)}\,{\rm erfc}\left(\frac{x}{2\sqrt{D_{\rm R'}t_2}}\right) \tag{S41}$$

$$\tilde{c}_{\rm O}^{(2)}(x,t_2) = \tilde{c}_{\rm O}^{\rm s(2)}{\rm erfc}\left(\frac{x}{2\sqrt{D_{\rm O}t_2}}\right) \qquad \tilde{c}_{\rm O'}^{(2)}(x,t_2) = \tilde{c}_{\rm O'}^{\rm s(2)}\,{\rm erfc}\left(\frac{x}{2\sqrt{D_{\rm O'}t_2}}\right)$$

so that the concentration profiles of the second pulse are given by:

Anodic compartment

$$c_{\rm R}^{(2)}(x,t) = c_{\rm R}^{*} + (c_{\rm R}^{\rm s(1)} - c_{\rm R}^{*})\,{\rm erfc}\left(\frac{x}{2\sqrt{D_{\rm R}\left(\tau_1+t_2\right)}}\right) + (c_{\rm R}^{\rm s(2)} - c_{\rm R}^{\rm s(1)}){\rm erfc}\left(\frac{x}{2\sqrt{D_{\rm R}t_2}}\right) \tag{S42}$$

$$c_{\rm O}^{(2)}(x,t) = c_{\rm O}^{\rm s(1)}{\rm erfc}\left(\frac{x}{2\sqrt{D_{\rm O}\left(\tau_1+t_2\right)}}\right) + (c_{\rm O}^{\rm s(2)} - c_{\rm O}^{\rm s(1)}){\rm erfc}\left(\frac{x}{2\sqrt{D_{\rm O}t_2}}\right)$$

Cathodic compartment

$$c_{\rm O'}^{(2)}(x,t) = c_{\rm O'}^{*} + (c_{\rm O'}^{\rm s(1)} - c_{\rm O'}^{*})\,{\rm erfc}\left(\frac{x}{2\sqrt{D_{\rm O'}\left(\tau_1+t_2\right)}}\right) + (c_{\rm O'}^{\rm s(2)} - c_{\rm O'}^{\rm s(1)})\,{\rm erfc}\left(\frac{x}{2\sqrt{D_{\rm O'}t_2}}\right) \tag{S43}$$

$$c_{\rm R'}^{(2)}(x,t) = c_{\rm R'}^{\rm s(1)}\,{\rm erfc}\left(\frac{x}{2\sqrt{D_{\rm R'}\left(\tau_1+t_2\right)}}\right) + (c_{\rm R'}^{\rm s(2)} - c_{\rm R'}^{\rm s(1)})\,{\rm erfc}\left(\frac{x}{2\sqrt{D_{\rm R'}t_2}}\right)$$

From expressions (S42) and (S43), together with the relationships between the current and the surface gradients of the redox species (eqns. (S13) and (S14)), the following expressions are immediately deduced for the interfacial concentrations:

Anodic compartment — Cathodic compartment

$$c_{\rm R}^{\rm s(2)} = c_{\rm R}^{*}\left[1-\left(I_{\rm 1,N}+\sqrt{\frac{t_2}{\tau_1+t_2}}\left(I_{\rm 2,N}-I_{\rm 1,N}\right)\right)\right] \qquad c_{\rm R'}^{\rm s(2)} = c_{\rm O'}^{*}\frac{\gamma'}{\varepsilon}\left(I_{\rm 1,N}+\sqrt{\frac{t_2}{\tau_1+t_2}}\left(I_{\rm 2,N}-I_{\rm 1,N}\right)\right)$$

$$c_{\rm O}^{\rm s(2)} = c_{\rm O}^{*}\frac{1}{\gamma}\left(I_{\rm 1,N}+\sqrt{\frac{t_2}{\tau_1+t_2}}\left(I_{\rm 2,N}-I_{\rm 1,N}\right)\right) \qquad c_{\rm O'}^{\rm s(2)} = c_{\rm O'}^{*}\left[1-\frac{1}{\varepsilon}\left(I_{\rm 1,N}+\sqrt{\frac{t_2}{\tau_1+t_2}}\left(I_{\rm 2,N}-I_{\rm 1,N}\right)\right)\right]$$

(S44)

and for the interfacial potentials:

$$E_{2,\mathrm{an}} = E^{0'}_{\mathrm{O/R}} + \frac{RT}{F}\ln\left(\frac{I_{1,\mathrm{N}} + \sqrt{\frac{t_2}{\tau_1+t_2}}\left(I_{2,\mathrm{N}} - I_{1,\mathrm{N}}\right)}{\gamma\left[1-\left(I_{1,\mathrm{N}} + \sqrt{\frac{t_2}{\tau_1+t_2}}\left(I_{2,\mathrm{N}} - I_{1,\mathrm{N}}\right)\right)\right]}\right) \tag{S45}$$

$$E_{2,\mathrm{cat}} = E^{0'}_{\mathrm{O'/R'}} + \frac{RT}{F}\ln\left(\frac{\varepsilon-\left(I_{1,\mathrm{N}} + \sqrt{\frac{t_2}{\tau_1+t_2}}\left(I_{2,\mathrm{N}} - I_{1,\mathrm{N}}\right)\right)}{\gamma'\left(I_{1,\mathrm{N}} + \sqrt{\frac{t_2}{\tau_1+t_2}}\left(I_{2,\mathrm{N}} - I_{1,\mathrm{N}}\right)\right)}\right) \tag{S46}$$

with:

$$I_{\mathrm{m,N}} = \frac{I_{\mathrm{i}}}{I_{\mathrm{lim,an}}\left(\tau_1+t_2\right)} \quad (\mathrm{m}=1,2) \tag{S47}$$

Taking into account eqn. (S5), the following relationship between the applied potential and the current is deduced:

$$E = E_{\mathrm{an}} - E_{\mathrm{cat}} = \Delta E^{0'} + \frac{RT}{F}\ln\left(\frac{\left(I_{1,\mathrm{N}} + \sqrt{\frac{t_2}{\tau_1+t_2}}\left(I_{2,\mathrm{N}} - I_{1,\mathrm{N}}\right)\right)^2}{\Gamma\left[\varepsilon-\left(I_{1,\mathrm{N}} + \sqrt{\frac{t_2}{\tau_1+t_2}}\left(I_{2,\mathrm{N}} - I_{1,\mathrm{N}}\right)\right)\right]\left[1-\left(I_{1,\mathrm{N}} + \sqrt{\frac{t_2}{\tau_1+t_2}}\left(I_{2,\mathrm{N}} - I_{1,\mathrm{N}}\right)\right)\right]}\right) \tag{S48}$$

so that, by analogy with (S23), it is deduced that:

$$\begin{aligned} f(\eta_2) &= I_{1,\mathrm{N}} + \sqrt{\frac{t_2}{\tau_1+t_2}}\left(I_{2,\mathrm{N}} - I_{1,\mathrm{N}}\right) \\ &= \frac{\left(\varepsilon+1\right)\Gamma e^{\eta_2} - \sqrt{\left(\varepsilon-1\right)^2\Gamma^2 e^{2\eta_2} + 4\varepsilon\,\Gamma e^{\eta_2}}}{2\left(\Gamma e^{\eta_2}-1\right)} \end{aligned} \tag{S49}$$

with:

$$\eta_2 = \frac{F(E_2 - \Delta E^{0'})}{RT} \tag{S50}$$

in such a way that the current response in the second potential pulse is given by:

$$I_2 = FA_{\mathrm{an}}c^*_{\mathrm{R}}\sqrt{\frac{D_{\mathrm{R}}}{\pi}}\left[\frac{1}{\sqrt{\tau_1+t_2}}f(\eta_1) + \frac{1}{\sqrt{t_2}}\left(f(\eta_2) - f(\eta_1)\right)\right] \tag{S51}$$

and the time-independent interfacial concentrations and interfacial potentials by:

Anodic compartment / Cathodic compartment

$$c_{\rm R}^{\rm s(2)} = c_{\rm R}^{*}\left(1 - f(\eta_2)\right) \qquad c_{\rm R'}^{\rm s(2)} = c_{\rm O'}^{*}\frac{f(\eta_2)\gamma'}{\varepsilon}$$

$$c_{\rm O}^{\rm s(2)} = c_{\rm R}^{*}\frac{f(\eta_2)}{\gamma} \qquad c_{\rm O'}^{\rm s(2)} = c_{\rm O'}^{*}\left(1 - \frac{f(\eta_2)}{\varepsilon}\right) \tag{S52}$$

$$E_{\rm 2,an} = E_{\rm O/R}^{0'} + \frac{RT}{F}\ln\left(\frac{f(\eta_2)}{\left(1 - f(\eta_2)\right)\gamma}\right) \qquad E_{\rm 2,cat} = E_{\rm O'/R'}^{0'} + \frac{RT}{F}\ln\left(\frac{\varepsilon - f(\eta_2)}{\gamma' f(\eta_2)}\right)$$

SI3. Any p-th potential pulse

When considering the application of successive potential pulses, E_1, E_2, … $E_{\rm p}$, the linearity of the problem enables us to propose that the solutions of the p-th pulse can be written as:

Anodic compartment / Cathodic compartment

$$c_{\rm R}^{\rm (p)}(x,t) = c_{\rm R}^{\rm (p-1)}(x,t) + \tilde{c}_{\rm R}^{\rm (p)}(x,t_{\rm p}) \qquad c_{\rm R'}^{\rm (p)}(x,t) = c_{\rm R'}^{\rm (p-1)}(x,t) + \tilde{c}_{\rm R'}^{\rm (p)}(x,t_{\rm p}) \tag{S53}$$

$$c_{\rm O}^{\rm (p)}(x,t) = c_{\rm O}^{\rm (p-1)}(x,t) + \tilde{c}_{\rm O}^{\rm (p)}(x,t_{\rm p}) \qquad c_{\rm O'}^{\rm (p)}(x,t) = c_{\rm O'}^{\rm (p-1)}(x,t) + \tilde{c}_{\rm O'}^{\rm (p)}(x,t_{\rm p})$$

where $c_{\rm i}^{\rm (p)}$ $({\rm i} \equiv {\rm O, R, O', R'})$ are the solutions of the p-th pulse and $c_{\rm R}^{\rm (p-1)}(x,t)$ the solutions of the previous pulse (p–1), which will be assumed to have the form of the solution deduced for the second pulse. Then, it is easily demonstrated that the bvp and so the solution for $\tilde{c}_{\rm i}^{\rm (p)}$ are identical to those of the second pulse. Therefore, by mathematical induction it is proven that the superposition principle applies [2], in such a way that the applied potential is given by:

$$E = E_{\rm an} - E_{\rm cat} = \Delta E^{0'} + \frac{RT}{F}\ln\left(\frac{\left(I_{\rm 1,N} + \sum_{\rm m=2}^{\rm p}\beta_{\rm m}\left(I_{\rm m,N} - I_{\rm m-1,N}\right)\right)^2}{\Gamma\left[\varepsilon - \left(I_{\rm 1,N} + \sum_{\rm m=2}^{\rm p}\beta_{\rm m}\left(I_{\rm m,N} - I_{\rm m-1,N}\right)\right)\right]\left[1 - \left(I_{\rm 1,N} + \sum_{\rm m=2}^{\rm p}\beta_{\rm m}\left(I_{\rm m,N} - I_{\rm m-1,N}\right)\right)\right]}\right) \tag{S54}$$

with:

$$\beta_{\rm m} = \sqrt{\frac{t_{\rm m,p}}{t_{\rm 1,p}}} \tag{S55}$$

$$t_{\rm m,p} = \begin{cases} \sum_{\rm j=m}^{\rm p-1}\tau_{\rm j} + t_{\rm p} & \text{if } {\rm m < p} \\ t_{\rm p} & \text{if } {\rm m = p} \end{cases} \tag{S56}$$

$\tau_{\rm j}$ being the duration of the j-th pulse, and the current response of the p-th pulse by:

$$I_{\rm p}=FA_{\rm an}c_{\rm R}^{*}\sqrt{\frac{D_{\rm R}}{\pi}}\sum_{\rm m=1}^{\rm p}\frac{1}{\sqrt{t_{\rm m,p}}}\left(f(\eta_{\rm m})-f(\eta_{\rm m-1})\right) \tag{S57}$$

where

$$f(\eta_0)=0$$
$$f(\eta_{\rm m>0})=\frac{(\varepsilon+1)\,\Gamma {\rm e}^{\eta_{\rm m}}-\sqrt{(\varepsilon-1)^2\,\Gamma^2{\rm e}^{2\eta_{\rm m}}+4\,\varepsilon\,\Gamma e^{\eta_{\rm m}}}}{2\left(\Gamma e^{\eta_{\rm m}}-1\right)} \tag{S58}$$

with

$$\eta_{\rm m}=\frac{F(E_{\rm m}-\Delta E^{0'})}{RT} \tag{S59}$$

Also, analogously to the first and second pulses, the following expressions hold for the interfacial concentrations and the interfacial potentials:

Anodic compartment

$$c_{\rm R}^{\rm s(p)}=c_{\rm R}^{*}\left(1-f(\eta_{\rm p})\right)$$
$$c_{\rm O}^{\rm s(p)}=c_{\rm R}^{*}\frac{f(\eta_{\rm p})}{\gamma}$$
$$E_{\rm p,an}=E_{\rm O/R}^{0'}+\frac{RT}{F}\ln\left(\frac{f(\eta_{\rm p})}{\left(1-f(\eta_{\rm p})\right)\gamma}\right)$$

Cathodic compartment

$$c_{\rm R'}^{\rm s(p)}=c_{\rm O'}^{*}\frac{f(\eta_{\rm p})\gamma'}{\varepsilon}$$
$$c_{\rm O'}^{\rm s(p)}=c_{\rm O'}^{*}\left(1-\frac{f(\eta_{\rm p})}{\varepsilon}\right) \tag{S60}$$
$$E_{\rm p,cat}=E_{\rm O'/R'}^{0'}+\frac{RT}{F}\ln\left(\frac{\varepsilon-f(\eta_{\rm p})}{\gamma' f(\eta_{\rm p})}\right)$$

References

(1) Molina, A.; González, J. *Pulse Voltammetry in Physical Electrochemistry and Electroanalysis*; Scholz, F., Ed.; Monographs in Electrochemistry; Springer International Publishing: Berlin, 2016. https://doi.org/10.1007/978-3-319-21251-7.

(2) Molina, A.; Serna, C.; Camacho, L. Conditions of Applicability of the Superposition Principle in Potential Multipulse Techniques: Implications in the Study of Microelectrodes. *J. Electroanal. Chem.* **1995**, *394* (1–2), 1–6. https://doi.org/10.1016/0022-0728(95)04005-9.
